# Prospective Associations of Tea Consumption With Risk of Cognitive Decline in the Elderly: A 1-Year Follow-Up Study in China

**DOI:** 10.3389/fnut.2022.752833

**Published:** 2022-02-21

**Authors:** Wei Li, Ling Yue, Shifu Xiao

**Affiliations:** ^1^Department of Geriatric Psychiatry, Shanghai Mental Health Center, Shanghai Jiao Tong University School of Medicine, Shanghai, China; ^2^Department of Geriatric Psychiatry, Alzheimer's Disease and Related Disorders Center, Shanghai Jiao Tong University, Shanghai, China

**Keywords:** tea, cognition, elderly, longitudinal study, corpus callosum

## Abstract

**Background:**

Previous studies show that the consumption of tea is associated with several beneficial outcomes for brain health, but there is little data among the elderly in China.

**Objective:**

The objective was to explore the longitudinal relationship between tea consumption and the risk of cognitive decline.

**Methods:**

The current data was obtained from the China Longitudinal Aging Study (CLAS), and a total of 3,246 residents aged 60 years and above were recruited in this study. Some of them (*N* = 111) underwent a standard T1-weighted magnetic resonance imaging (MRI), from which the volumes of the corpus callosum (CC) and hippocampus were calculated, and detailed tea consumption information was obtained through a standardized questionnaire at baseline. The cognitive diagnosis of each participant was made by attending psychiatrists at baseline and follow-up. Their overall cognitive function was assessed by the Montreal Cognitive Assessment (MoCA), while their associative learning ability was assessed by an associative learning test (ALT). Finally, 1,545 elderly with normal cognitive function completed the baseline and follow-up assessment and were included in the final study.

**Results:**

After controlling gender, education, smoking, take exercise and hobbies, we found that the elderly with tea consumption habits had a lower incidence rate of cognitive decline (*p* = 0.002, OR = 0.604, 95%CI:0.437~0.836) and tea consumption was negatively correlated with the change scores of MoCA (r = −0.056, *p* = 0.029). What's more, the CC_posterior volume of tea drinkers was significantly smaller than that of non-tea drinkers, while the baseline ALT score of tea drinkers was significantly higher than that of non-tea drinkers. The results of correlation analysis showed that the CC_posterior volume was significantly correlated with ALT change score (r = −0.319, *p* = 0.010).

**Conclusions:**

The habit of tea consumption is associated with less incidence of cognitive impairment among the Chinese elderly, and it may prevent a decline in memory and associative learning by affecting the volume of the posterior corpus callosum.

## Introduction

Tea, a beverage prepared from the leaves of Camellia sinensis, has been consumed extensively in China (1). Accumulating evidence (2–4) shows that the consumption of tea (including green, oolong, and black varieties) is associated with several beneficial outcomes for brain health (such as lower levels of depression). It was not until recent years, however, the effect of tea consumption on mood and mental performance has been increasingly investigated (5). For example, Schimidt et al. (6) found that eight weeks of green tea supplementation before the ischemia-reperfusion event showed a neuroprotective effect in male rats. Altermann et al. (7) also found that short-term tea supplementation showed a neuroprotective role, attenuated redox imbalance, and might have a beneficial impact on cognitive function after stroke. Although substantial evidence from animal studies and *in vitro* has indicated that tea preparations exert neuroprotective activities, the possible preventive effect of tea consumption against incident cognitive impairment in humans has remained unclear because of the lack of epidemiologic studies.

Until now, there were only three longitudinal studies involving tea consumption and cognitive change in China. For example, Zeng et al. (8) found that tea consumption was significantly associated with a lower risk of cognitive disability at advanced ages and interacted with the FOXO genotypes. Feng et al. (9) found that tea consumption was associated with better cognitive performance in community-living Chinese older adults and the protective effect of tea consumption on cognitive function was not limited to a particular type of tea. However, another longitudinal study conducted in China showed that black and oolong teas were associated with lower risks of cognitive impairment, but depended on frequency (10). So their conclusions were not completely consistent. What's more, all of the above studies used neuropsychological tests as the diagnostic basis of cognitive state, so it was likely to cause evaluation errors.

Since there are no pharmacologic treatments proven to cure or slow the progression of dementia, it is important to determine the reversible factors. Therefore, we conducted a one-year follow-up study to explore the effects of tea consumption type, frequency, and duration on cognitive function of the elderly with normal cognitive function, and to explore the possible imaging mechanism by using the structural magnetic resonance imaging (MRI).

## Materials and Methods

### Participants

**Figure 1:**
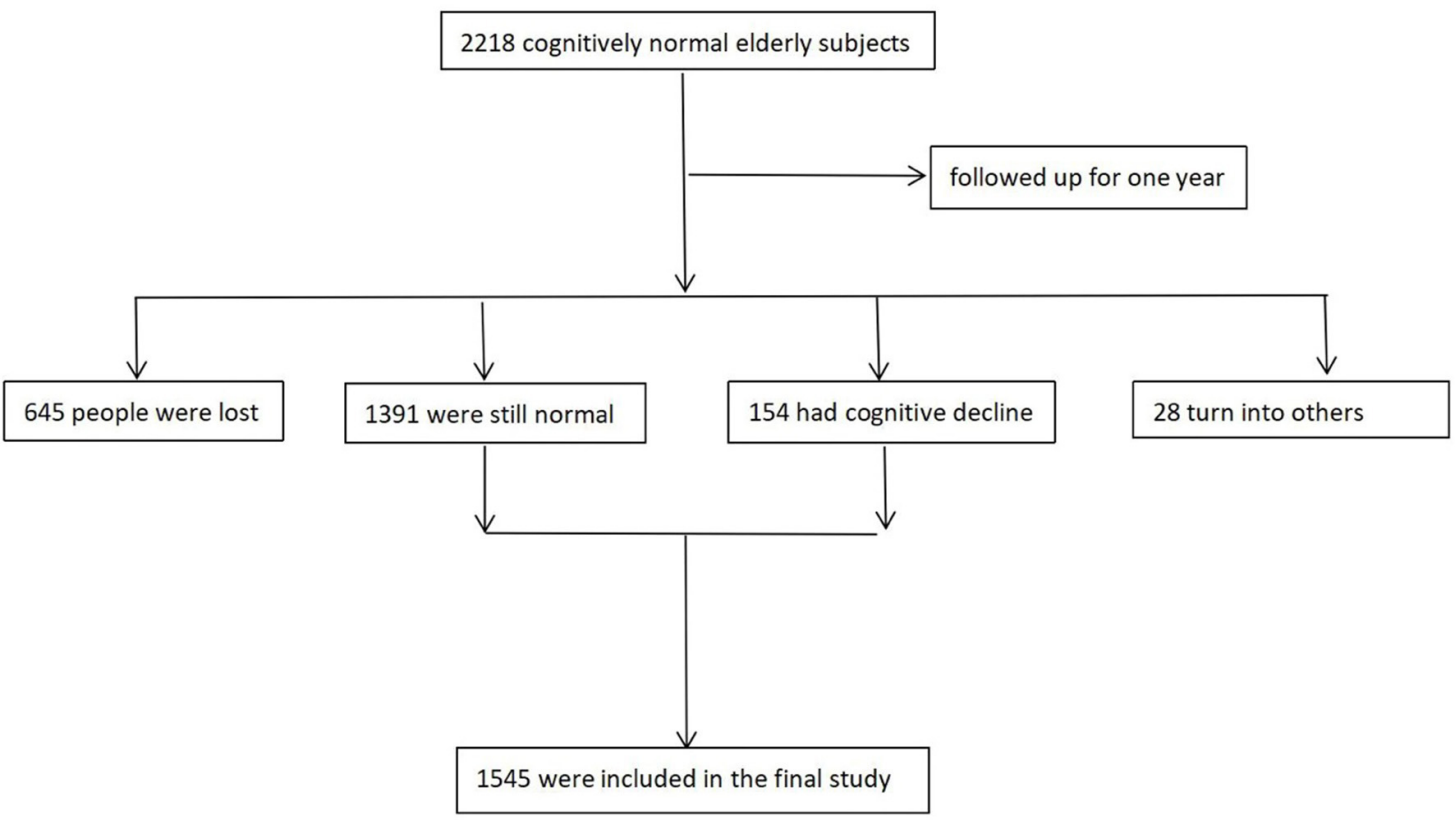
Research flow chart of the whole study.

The design of the China Longitudinal Aging Study (CLAS) (11) has been described in detail elsewhere, which represents a survey of community-based epidemiological studies in China. In brief, twenty target communities (i.e., 2 rural and 18 urban) located in the western, eastern and mid parts of China had been included. According to the 2010 national census, the population aged 60 and over was included in a database. A simple random sample comprising 3,246 residents was selected to identify potential participants. Among them, 2,267 completed both the baseline and one-year follow-up assessments, and then we selected those elderly with normal cognition at baseline as the study subjects. Finally, 1,545 old people with normal cognition entered the final study. Figure 1 presents the research flow.

All the subjects had signed informed consent before this study was initiated and ethical approval was obtained from the Ethics Committee of Shanghai Mental Health Center, and the ethical code was 2011-YJ-14.

### Clinical Assessment

All the participants underwent a screening process that included a review of their medical history, physical and neurological examinations, laboratory tests, and magnetic resonance imaging (MRI) scans. Mild cognitive impairment (MCI) was diagnosed according to the revised Petersen's diagnostic algorithm (12): (1) memory complaint; (2) objective memory impairment; (3) absence of dementia; (4) preservation of general cognitive function and intact activities of daily living. Dementia (including Alzheimer's disease, vascular dementia, and mixed dementia) was diagnosed according to the Diagnostic and Statistical Manual of Mental Disorders, fourth edition. All the diagnoses were performed by trained and qualified medical clinicians.

### Neuropsychological Tests

In the current study, the Montreal Cognitive Assessment (MoCA) (13) was used to assess participants' overall cognitive function at baseline and during follow-up, and the associative learning test (ALT) was used to assess their associative learning ability (14). Since depression could affect cognitive performance, in the current study, we excluded severe depression by using the Geriatric Depression Scale (GDS) (15).

### Tea Consumption

Detailed information on tea consumption (including the type, frequency, and duration of tea consumption) was collected by using the food-frequency questionnaire (FFQ) (16) at baseline. The questions were designed according to the habitual intake of common types of teas among local elderly by using indigenous terms and references: green tea, red tea, oolong tea, and others. The frequency of consumption of each type of tea was assessed by the four-category question, “During the past month, how many times a week do you drink tea in general (occasionally, 1–3 days a week, 4–6 days a week, every day)”. And the duration of tea consumption was assessed by this question, “How long did you drink tea”.

### Covariates

General demographic information (including age, gender, education), daily living information (including smoking history, consumption of alcohol, physical activities, and hobby), as well as disease information (such as hypertension and diabetes) were collected by a standardized questionnaire. And those variables that differed between tea consumption and non-tea consumption groups were considered as covariates.

### Structural Magnetic Resonance

The brain structure image was acquired by using a Siemens Magnetom Verio 3.0 T scanner (Siemens, Munich, Germany). The parameters of T1-weighted 3D magnetization prepared rapid gradient echo (MPRAGE) sequences were as follows: TE = 2.98 ms, TR = 2,300 ms; matrix size = 240 × 256; flip angle of 9 degree, field of view (FOV) = 240 × 256 mm; slice thickness = 1.2 mm. Volumetric data was assessed by automated procedures, which have been described by Wolz et al. (17). For each subject, volume and asymmetry with hippocampus, corpus callosum (CC), as well as the brain size index were extracted (by using FreeSurfer). Moreover, to assess the role of differences in left and right, an asymmetry index was computed using the equation: [right volume-left volume]/[total volume] × 100%. Quality control was carefully conducted by overlapping the output parcellations on FreeSurfer's template and visual assessment was performed to ensure the registration and parcellation quality.

### Follow-Up (Incident Cognitive Impairment)

All individuals (*n* = 1,545) included in the final study were assessed for baseline and follow-up. In tea drinking group (*n* = 793), 50 transformed into amnesic mild cognitive impairment (aMCI), 6 into vascular mild cognitive impairment (vMCI), 3 into Aizheimer's disease (AD), 5 into vascular dementia (VD), 2 into mixed dementia (MD). And in non-tea drinking group (*n* = 752), 61 transformed into aMCI, 9 into vMCI, 1 into subjective cognitive impairment (SCI), 8 into AD, 5 into VD, 4 into MD. Then we assigned all cases of cognitive decline (including aMCI, vMCI, SCI, AD, VD, and MD) into the cognitive decline group.

## Statistical Analysis

Continuous variables were expressed as mean ± standard deviation (SD), and categorical variables were expressed as frequencies (%). To test whether data conforms to normal distribution, we applied a single sample Kolmogorov-Smirnov test. Next, we used independent sample *t*-test and Kruskal-wallis H to compare the normal data and non-normal data between the tea drinking group and the non-tea drinking group, respectively. And we also used Chi-square tests to compare those classification variables. Then Cox regression analysis was used to further explore the relationship between tea consumption and cognitive change (controlled for other relevant variables) and partial correlation analysis was used to explore the correlation between tea consumption and the change of MoCA scores. Two-tailed tests were used at a significance level of P <0.05 for all analyses. The data was analyzed using SPSS 22.0 (IBM Corporation, Armonk, NY, USA).

## Results

### Characteristic of Subjects With Different Tea Consumption Habits

Overall, older people with tea consumption habits had a lower rate of cognitive decline (Pearson x2 = 4.912, *p* = 0.027), however, it had nothing to do with the type, frequency, or duration of tea consumption. There were statistical differences (*p* < 0.05) in education, gender, smoker, take exercise, hobby and follow-up scores of MoCA between the tea consumption group and non-tea-consumption group, while no statistical difference (*p* > 0.05) was found in age, baseline scores of MoCA, GDS, drinker, napping, hypertension, and diabetes between the two groups. Table 1 shows the results.

**Table 1:** Baseline characteristics by tea consumption in 1,545 older people.

**Characteristics**	**Tea drinker** **(*n* = 793)**	**Non-tea drinker** **(*n* = 752)**	**X^**2**^ OR T**	**P**
Age, y	70.14 ± 7.68	70.55 ± 7.43	−1.005	0.315
Education, y	5.96 ± 3.43	7.88 ± 3.26	−11.24	<0.001^*^
Baseline MoCA	23.27 ± 4.85	22.85 ± 5.18	1.621	0.105
Follow-up of MoCA	23.51 ± 5.01	22.64 ± 5.70	3.181	0.001^*^
MoCA change value	0.22 ± 3.24	−0.21 ± 3.97	2.306	0.021^*^
GDS	3.62 ± 3.86	3.75 ± 4.06	−0.667	0.505
**Type of tea**				
Green tea, *n* (%)	478 (60.3)			
Red tea, *n* (%)	68 (8.6)			
Oolong tea, *n* (%)	20 (2.5)			
Scented tea, *n* (%)	105 (13.2)			
Two or more, *n* (%)	122 (15.4)			
**The frequency of drinking tea**				
Occasionally	69 (8.7)			
1–3 times a week	75 (9.5)			
4–6 times a week	48 (6.1)			
Everyday	601 (75.8)			
Duration, y	29.71 ± 17.25			
Male, *n* (%)	499 (62.9)	253 (33.6)	132.474	<0.001^*^
Smoker, *n* (%)	331 (41.7)	112 (14.9)	136.023	<0.001^*^
Drinker, *n* (%)	373 (47.0)	356 (47.3)	0.014	0.919
Take exercise, *n* (%)	623 (78.6)	549 (73.0)	6.508	0.012^*^
Hobby, *n* (%)	539 (68.0)	461 (61.3)	7.513	0.007^*^
Napping, *n* (%)	460 (58.0)	420 (55.9)	0.732	0.411
Hypertension, *n* (%)	373 (47.0)	356 (47.3)	0.014	0.919
Diabetes, *n* (%)	114 (14.4)	114(15.2)	0.188	0.668

*MoCA, Montreal Cognitive Assessment; GDS, Geriatric depression scale;*

* *p < 0.05*.

### The Results of the Multiple Cox Regression Model

**Figure 2:**
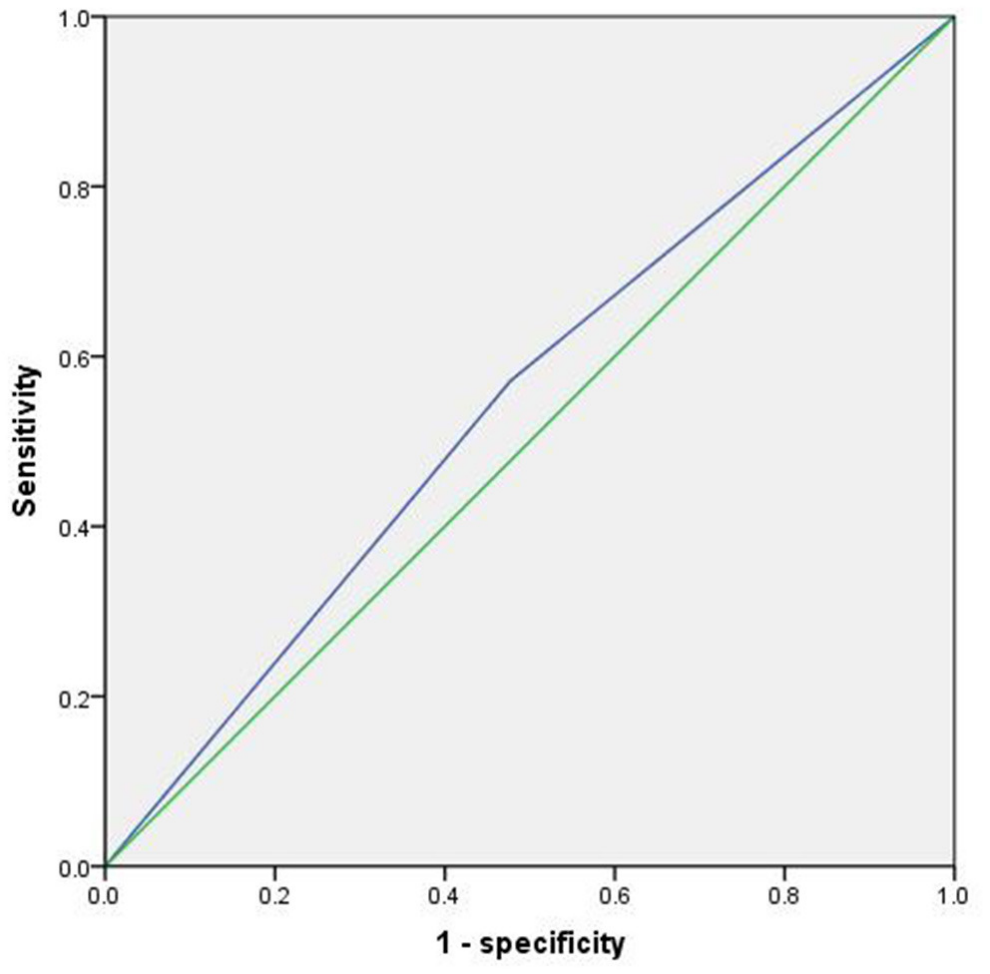
ROC curve of tea consumption predicting cognitive decline.

Multiple Cox regression model was used to explore the relationship between tea consumption and future cognitive decline (Cognitive decline was regarded as the dependent variable, and transition time was taken as the time variable). Model 1 did not control any variables, and the results showed that tea consumption was a protective factor for cognitive decline (*p* = 0.001, OR = 0.580, 95%CI: 0.419~0.801); Model 2 controlled some variables, such as gender and education, and Model 3 furtherly controlled other variables, such as smoking, take exercise and hobbies, and different statistical models still do not change the statistical results (Table 2). Then the ROC curve was used to explore the sensitivity and specificity of tea consumption to predict cognitive decline, and the area under the curve was 0.547 (*p* = 0.055, 95%CI: 0.499~0.595). Figure 2 shows the results.

**Table 2:** Results of COX regression analysis.

**Variables**	**B**	**S.E**	**Wald**	**df**	**p**	**HR**	**95% confidence interval**	
**Model 1**								
Tea drinker	−0.545	0.165	10.905	1	0.001^*^	0.580	0.419	0.801
**Model 2**								
Tea drinker	−0.502	0.174	8.324	1	0.004^*^	0.606	0.431	0.851
Education	−0.413	0.072	33.390	1	<0.001^*^	0.662	0.575	0.761
Male	−2.993	0.486	37.852	1	<0.001^*^	0.050	0.019	0.130
**Model 3**								
Tea drinker	−0.423	0.178	5.671	1	0.017^*^	0.655	0.462	0.928
Education	−0.354	0.076	21.596	1	<0.001^*^	0.702	0.604	0.815
Male	−2.477	0.523	22.475	1	<0.001^*^	0.084	0.030	0.234
Smoking	−0.244	0.243	1.013	1	0.314	0.783	0.487	1.261
Take exercise	−0.149	0.177	0.704	1	0.401	0.862	0.609	1.219
Hobby	−0.365	0.179	4.178	1	0.041^*^	0.694	0.489	0.958

*Model 1 contains only tea drinking; Model 2 contains tea drinking, gender and education; Model 3 contains tea drinking, gender, education, smoking, take exercise and hobbies*.

* *p < 0.05*.

### The Results of Partial Correlation Analysis

**Figure 3:**
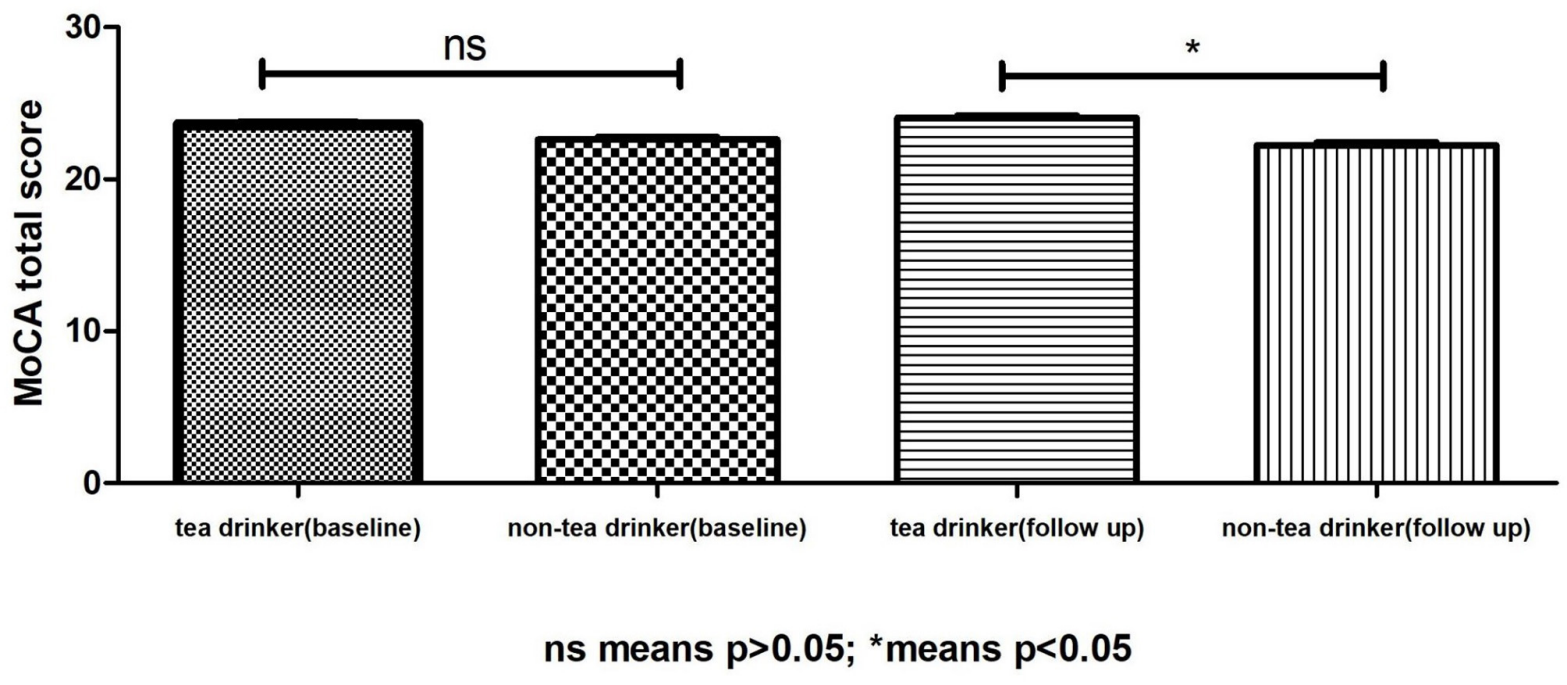
Trends of MOCA scores between tea drinkers and non-tea drinkers.

Although there was no difference in the baseline MoCA scores between the tea-consumption group and non-tea consumption group, the MoCA score of the tea-consumption group was significantly higher than that of the non-tea consumption group after 1 year (Figure 3). After controlling for gender, baseline MoCA scores, education, exercise and hobbies, it was found that tea consumption was negatively correlated with the changes score of MoCA (r = −0.056, *P* =0.029).

### The Interaction of Hobbies and Gender With Tea Consumption

Since there was a higher proportion of males and hobbies in the tea drinking group, further stratification was performed to exclude the effects of gender and hobbies. By using Chi-square tests, we found that among men, tea consumption had a lower risk of future cognitive impairment (6.6 vs 11.2%, X^2^ = 5.156, *p* = 0.032), while among women, tea consumption had no effect (9.9 vs 12.6%, X^2^ = 1.223, *p* = 0.283) on the incidence of future cognitive impairment. Using the same method, we found that tea consumption was a protective factor for cognitive decline regardless of whether a hobby existed (6.3 vs 12.6%, X^2^ = 8.953, *p* = 0.004; 7.6 vs 18.2, X2 = 19.475, *p* < 0.001).

### The Results Associated With Magnetic Resonance

**Figure 4:**
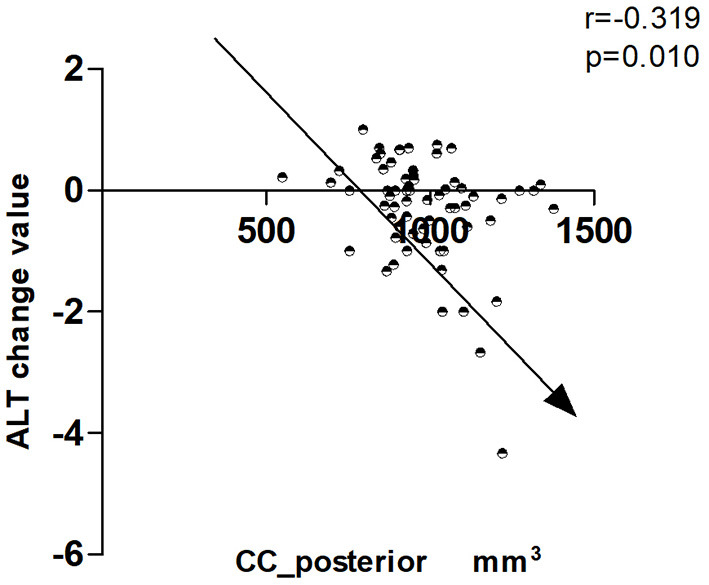
Correlation between CC_posterior and change value in ALT.

To explore the possible mechanism of tea drinking affecting cognitive function, in this part, we randomly selected 111 people (tea drinker, *n* = 54; non-tea drinker, *n* = 57), all of whom completed MRI. The random method is to use the SPSS's random number generator. No statistical difference (*p* > 0.05) was found in age, education, males, smokers, drinkers, take exercise, hobbies, hypertension, diabetes, total brain volume, left hippocampus volume, right hippocampus volume, CC_anterior volume, CC_Central volume, baseline MoCA score, MoCA score during follow-up, MoCA change value, ALT score during follow-up, and ALT change value between the two groups. However, the CC_posterior volume of tea drinkers was significantly smaller than that of non-tea drinkers, while the baseline ALT score of tea drinkers was significantly higher than that of non-tea drinkers (Table 3). The results of correlation analysis showed that the CC_posterior volume was significantly correlated with ALT change score (r = −0.319, *p* = 0.010), suggesting that drinking tea may prevent cognitive decline (especially associative learning and memory) by affecting the CC_posterior volume. Figure 4 presents the results.

**Table 3:** Effects of tea drinking on cognitive function and cognition-related brain regions.

**Characteristics**	**Tea drinkers** **(*n* = 54)**	**Non-tea drinkers** ***n* = 57)**	**T**	**P**
Age, y	69.67 ± 7.697	71.28 ± 8.079	−1.076	0.284
Education, y	9.08 ± 4.300	8.61 ± 4.218	0.564	0.574
Male, *n* (%)	35 (64.8)	37 (64.9)	1.000	0.574
Smoker, *n* (%)	23 (42.6)	16 (28.1)	2.566	0.117
Drinker, *n* (%)	15 (27.8)	8 (14.0)	3.188	0.101
Take exercise, *n* (%)	32 (59.3)	34 (59.6)	0.002	1.000
Hobby, *n* (%)	37 (68.5)	29 (50.9)	3.580	0.082
Hypertension, *n* (%)	20 (37.0)	21 (36.8)	0	1.000
Diabetes, *n* (%)	7 (13.0)	6 (10.5)	0.159	0.733
**Brain structure**				
Total brain volume, cm^3^	1485.44 ± 131.605	1475.91 ± 135.964	0.375	0.709
Center hippocampus, mm^3^	3676.10 ± 384.162	3574.24 ± 472.829	1.242	0.217
Right hippocampus, mm^3^	3852.21 ± 451.510	3783.28 ± 499.481	0.761	0.448
CC_anterior, mm^3^	854.76 ± 146.967	879.43 ± 145.036	−0.890	0.375
CC_Central, mm^3^	451.85 ± 102.669	466.18 ± 100.002	−0.745	0.458
CC_posterior, mm^3^	950.42 ± 181.166	1023.41 ± 172.956	−2.172	0.032^*^
**Neuropsychological tests**				
Baseline MoCA	24.66 ± 3.937	23.86 ± 4.228	1.026	0.307
Follow-up of MoCA	23.61 ± 4.451	23.22 ± 5.357	0.317	0.752
MoCA change value	0.008 ± 0.155	−0.049 ± 0.236	1.148	0.256
Baseline ALT	6.92 ± 3.142	5.68 ± 3.216	2.044	0.043^*^
Follow-up of ALT	6.80 ± 3.008	6.13 ± 4.647	0.700	0.486
ALT change value	−0.198 ± 0.738	−0.404 ± 1.023	0.932	0.355

* *p < 0.05; CC means corpus callosum; ALT, Associative learning test*.

## Discussion

As far as we know, this was the largest longitudinal study involving the relationship between tea consumption and the incidence of cognitive impairment in China. This study demonstrated that tea consumption was significantly associated with a decreased risk of cognitive decline (dementia or MCI), regardless of the frequency, type, or duration of tea consumption. What's more, we found that drinking tea may prevent cognitive decline (especially associative learning and memory) by affecting the volume of the posterior corpus callosum.

In the current study, we investigated the tea consumption habits (such as type, frequency, and duration) of the elderly with normal cognitive function by using standardized questionnaires. During the follow-up period, 8.3% of the tea consumption group had a cognitive decline, which was lower than that (11.3%) of the non-tea consumption group. It is worth noting that there were more men in the tea-drinking group and a higher proportion of people who had hobbies. Previous studies have shown that both male sex and hobbies are protective factors for cognitive decline (18–21). In order to eliminate the influence of gender and hobbies on the results, we adopted two methods to verify, the first was a multivariate step-by-step COX regression model: Model 1 contains only tea drinking; Model 2 contains tea drinking, gender and education; Model 3 contains tea drinking, gender, education, smoking, take exercise and hobbies. Ultimately, we found that tea consumption was a protective factor for cognitive decline, regardless of which model we used. The second was the interactive Chi-square test, we found that among men, tea consumption had a lower risk of future cognitive impairment, while among women, tea consumption had no effect on the incidence of future cognitive impairment. However, for hobbies, tea consumption was a protective factor for cognitive decline in older adults regardless of whether hobbies existed.

Next, we specifically discussed the influence of tea type, frequency, and duration on cognitive function, and found that the three had nothing to do with cognitive changes (*p* > 0.05). Feng et al. (22) found that regular tea consumption was associated with a lower risk of neurocognitive disorders among the Chinese elderly. In Godos J et al.'s study, they also found that higher intakes of total phenolic acids and hydroxycinnamic acids were significantly inversely associated with cognitive impairment (23). Lenore Arab et al. (2) found that tea consumption prevented cognitive function only in older women, but not men. In Yoshiro Shirai et al.'s study, they found that drinking green tea might prevent cognitive decline in older Japanese (24). In Noguchi-Shinohara M et al.' s study, they found that consumption of green tea, but not black tea or coffee, was associated with reduced risk of cognitive decline (25). Therefore, our conclusions were partially consistent, and the differences were mainly due to the investigation methods, as we used the clinical diagnosis as the basis for diagnosis, while they used the Mini-Mental State (MMSE). Although MMSE is the most commonly used cognitive assessment tool, it is not highly sensitive and has a ceiling and floor effect (26). In order to overcome the limitations of previous studies, we replaced the neuropsychological assessment with clinical assessment and added MRI, thus greatly improving the diagnostic accuracy.

To explore the biological mechanism of tea consumption to prevent cognitive decline, we evaluated the baseline MRI of some elderly people, and finally found that tea drinkers might be able to prevent cognitive decline by influencing the volume of the posterior corpus callosum (CC). The corpus callosum is the largest white matter tract in the brain, containing upwards of 200 million axons, which is also an important structure for several neurological functions, including attention processing, social function, emotional processing, integration of lateralized sensory input, and regulation of higher-order cognitive (27, 28). Many studies have shown that AD is associated with CC atrophy, especially in the posterior part (29), but the exact pattern of subregional CC atrophy at different stages of the disease is unclear. In Kristian Steen Frederiksen's study (30), he found that early AD atrophy occured mainly in the posterior part of CC, and CC atrophy was associated with faster disease progression. Therefore, the posterior corpus callosum (CC) atrophy might be the neuroanatomical basis of AD memory loss. In addition, a recent texture analysis also demonstrated the relationship between CC and AD (31). In our study, we found that tea consumption was primarily associated with the volume of CC_posterior and may prevent cognitive decline through this site. Wang et al. (32) found that green tea consumption could impact brain activity during resting state. Carmichael et al. (33) found that one-month supplementation with green tea catechins was associated with suggestive changes in cognitive functioning as well as brain functional connectivity and modification of brain activation in cognitively healthy older adults. Since there was no study on tea consumption and structural magnetic resonance imaging, we could not judge whether our conclusions were consistent.

Several other mechanisms can also explain why tea consumption may reduce the risk of cognitive impairment: in animal studies, M. He et al. found that tea drinking could reduce the accumulation of Aβ and released neuronal injury in the hippocampus of AD mode mice (34); K. Rezai-Zadeh, G.W found that tea could regulate the tau protein profile and markedly suppress the phosphorylated tau isoforms (35); A.L. Lardner et pointed that tea could exert relaxant and calming effects, while simultaneously increasing alertness (36); Todorova et al. reported gastro-protective and anti-inflammatory effects *in vitro* alongside a correlation between the phenolic content of the tea extract and antioxidant activity (37); In human experiments, tea has also been shown to increase levels of the neurotrophic brain-derived neurotrophic factor (BDNF) (38). Moreover, polyphenols are the major active compounds present in teas, which have been proved to have beneficial effects against several pathological diseases including diabetes, cancer, and cardiovascular diseases (39). Most current research on the benefits of tea polyphenols mainly focuses on the following aspects, such as its antioxidation (40), hormesis (41), anti-inflammatory effects (42), re-allocation of energy (43), as well as DNA repair and conservation (44). We speculate that it is the interaction of these mechanisms that leads to the cognitive protective effect of consumption.

We have to admit that there are two limitations to our study. First, information on tea consumption was obtained by self-reporting rather than objectively assessed, thus the possibility of recall bias existed. Second, short follow-up time was also a major limitation of our study.

## Conclusions

In conclusion, the habit of tea consumption is associated with less incidence of cognitive impairment among the Chinese elderly, and the protective effect of tea consumption on cognitive function is not limited to a particular type or frequency of tea. What's more, tea consumption may prevent cognitive decline (especially associative learning and memory) by affecting the volume of the posterior corpus callosum.

## Data Availability Statement

The original contributions presented in the study are included in the article/Supplementary Material, further inquiries can be directed to the corresponding authors.

## Ethics Statement

The studies involving human participants were reviewed and approved by the Ethics Committee of Shanghai Mental Health Center. The patients/participants provided their written informed consent to participate in this study. Written informed consent was obtained from the individual(s) for the publication of any potentially identifiable images or data included in this article.

## Author Contributions

WL and LY contributed to the study concept and design. SX analyzed the data and drafted the manuscript. WL directed the analysis and statistics of MRI data. All authors read and approved the final manuscript.

## Funding

This study was supported by grants from the clinical research center project of Shanghai Mental Health Center (CRC2017ZD02), Shanghai Clinical Research Center for Mental Health (19MC1911100), the Cultivation of Multidisciplinary Interdisciplinary Project in Shanghai Jiaotong University (YG2019QNA10), curriculum reform of Medical College of Shanghai Jiaotong University, and the Feixiang Program of Shanghai Mental Health Center (2020-FX-03). This project was also funded by the Shanghai Elderly Brain Health Cohort Institute, Chinese Academy of Sciences (XDA12040101), Shanghai Clinical Research Center for Mental Health (SCRC-MH, 19MC1911100), the National Natural Science Foundation of China (82001123 and 82101564), the Shanghai Science and Technology Committee (20Y11906800), and the Feixiang Program of Shanghai Mental Health Center (2018-FX-05). We also thank for the supportive of Shanghai brain health foundation (SHBHF2016001).

## Conflict of Interest

The authors declare that the research was conducted in the absence of any commercial or financial relationships that could be construed as a potential conflict of interest.

## Publisher's Note

All claims expressed in this article are solely those of the authors and do not necessarily represent those of their affiliated organizations, or those of the publisher, the editors and the reviewers. Any product that may be evaluated in this article, or claim that may be made by its manufacturer, is not guaranteed or endorsed by the publisher.

## Abbreviations

aMCI: Amnesic mild cognitive impairment
vMCI: Vascular mild cognitive impairment
AD: Alzheimer's disease
VD: Vascular dementia
MD: Mixed dementia
MMSE: Mini-Mental State
MoCA: Montreal Cognitive Assessment
GDS: Geriatric Depression Scale
CLAS: China Longitudinal Aging Study
MRI: Magnetic resonance imaging
BDNF: Neurotrophin brain-derived neurotrophic factor
CC: Corpus callosum
FFQ: Food-frequency questionnaire.
